# Late-Onset Isolated Growth Hormone Deficiency

**DOI:** 10.1210/jcemcr/luad011

**Published:** 2023-03-03

**Authors:** Julie G Samuels, Sri Nikhita Chimatapu, Martin O Savage, Robert Rapaport

**Affiliations:** Division of Pediatric Endocrinology and Diabetes, Icahn School of Medicine at Mount Sinai, New York, NY 10029, USA; Division of Pediatric Endocrinology, UCLA Mattel Children's Hospital, Los Angeles, CA 90095, USA; William Harvey Research Institute, Barts and the London School of Medicine & Dentistry, Queen Mary, University of London, London E1 2AD, UK; Division of Pediatric Endocrinology and Diabetes, Icahn School of Medicine at Mount Sinai, New York, NY 10029, USA

**Keywords:** growth, growth hormone stimulation test, growth hormone deficiency, idiopathic short stature

## Abstract

Two male patients, who presented at 13.5 and 13.9 years of age with growth failure and short stature, were ultimately diagnosed with isolated growth hormone deficiency (GHD). Patient 1 was first evaluated when his height declined from −0.67 SD to −1.3 SD. He had a peak growth hormone (GH) concentration to GH stimulation test (GHST) of 16.9 ng/mL (16.9 μg/L) and remained untreated. As puberty advanced, his height decreased further to −1.65 SD. A second GHST while his serum testosterone was 79 ng/dL (2.74 nmol/L) had a peak GH of 5.4 ng/mL (5.4 μg/L), consistent with GHD. He was treated with GH for 4.8 years and reached adult height of 180.5 cm (0.57 SD), gaining 2.22 SDS. Patient 2, height −2.63 SD, had an unstimulated peak GH concentration of 19 ng/mL (19 μg/L). As puberty advanced, his height decreased further to −2.96 SD. Repeat peak GH concentration was 9.2 ng/mL (9.2 μg/L) when serum testosterone was 83.9 ng/dL (2.91 nmol/L). GH treatment resulted in rapid increase of height velocity from 1.8 cm/year to 11.3 cm/year in 6 months, consistent with GHD. Both patients demonstrate that GHD may develop over time and cannot be excluded by a single GHST. Longitudinal monitoring of children with poor growth as puberty progresses is essential to uncover GHD.

## Introduction

To investigate possible growth hormone deficiency (GHD), patients with short stature, low height velocity (HV), and low growth factor levels for age and pubertal stage undergo a GH stimulation test (GHST). Although flawed, current guidelines use GHST as essential for diagnosing GHD, with the peak GH levels below 10 ng/mL considered consistent with GHD [1–3].

GHD can be congenital or emerge later in life. Patients with organic central nervous system lesions such as septo-optic dysplasia or craniopharyngioma have been shown to gradually develop GHD [4]. Gradual development of GHD in children without organic pathology has been rarely documented. Zadik et al followed 3 patients with normal results on GHST who underwent retesting 3 to 4 years later due to growth deceleration. All 3 patients had peak responses below 10 ug/mL upon reevaluation, suggesting developing GHD [5]. Other pituitary hormone deficiencies including thyrotropin (thyroid-stimulating hormone; TSH), adrenocorticotropic hormone (ACTH), and gonadotropins may also develop over time in patients with apparent isolated GHD [6].

We present 2 patients evaluated for growth failure and short stature in whom GHD was only diagnosed after longitudinal follow-up and repeat GHST. Without such evaluations, incorrect diagnoses such as constitutional delay of growth and puberty or idiopathic short stature would have been considered.

## Case Presentation

### Case 1

**Figure 1.**
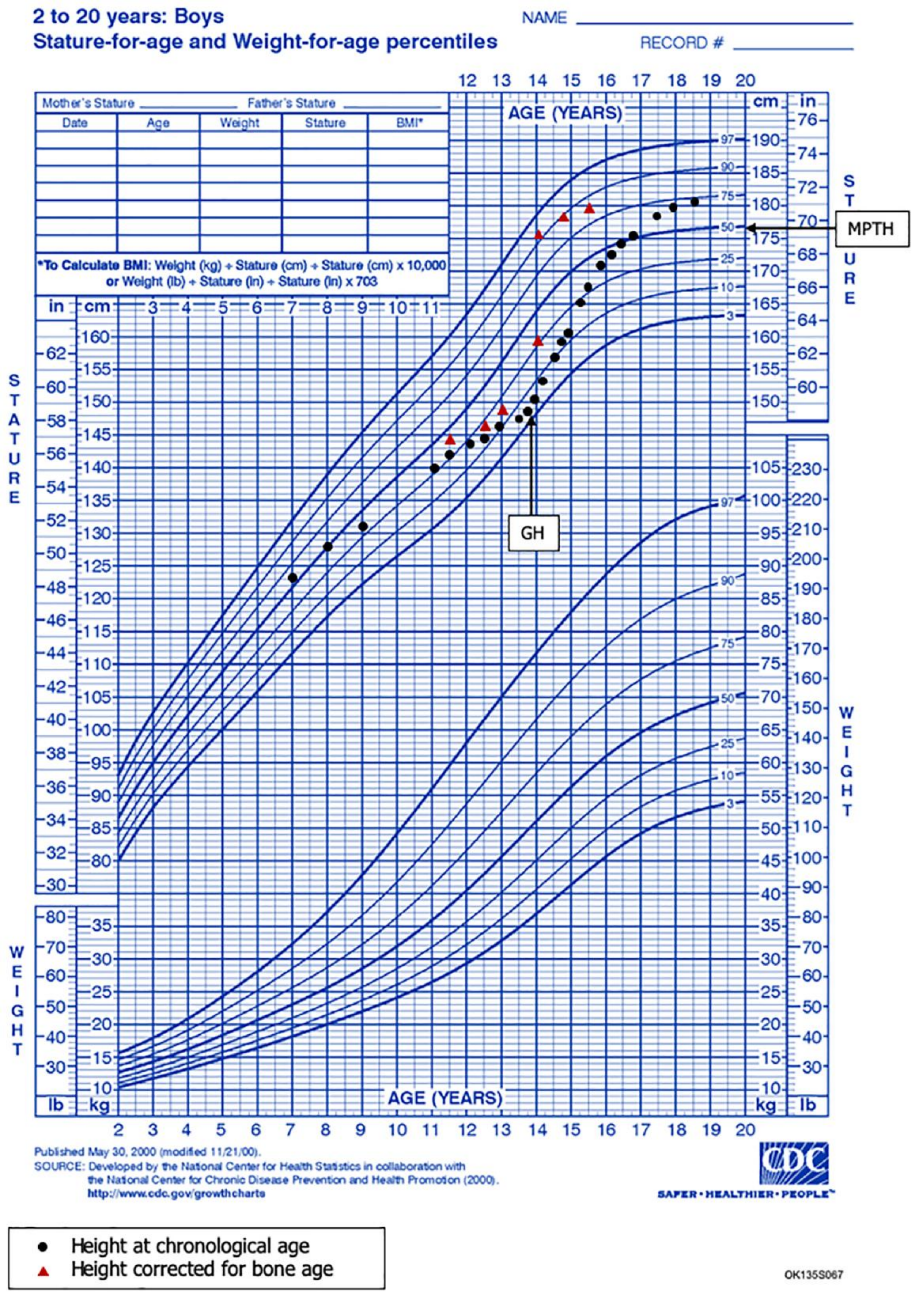
Patient 1 Growth Chart. Longitudinal growth chart in patient 1 before and after growth hormone treatment. GH, growth hormone; MPTH, Mid-Parental Target Height.

The patient was initially evaluated at 11.5 years at another institution. His height had decreased from the 50th (0 SD) to the 25th percentile (−0.67 SD; Fig. 1). He had no pertinent family history. His mid-parental target height was 176.7 cm at 50th percentile (0 SD). His body mass index (BMI) was on the 40th percentile, and he was prepubertal. His insulin-like growth factor 1 (IGF-1) level was 105 ng/mL (13.7 nmol/L; −1.68 SD for Tanner stage I) and his bone age was delayed by 6 months. His height declined to the 10th percentile (−1.3 SD) and an unprimed GHST with arginine and clonidine at age 13 showed a peak GH concentration of 16.9 ng/mL (16.9 μg/L; Table 1).

**Table 1.** Patient 1

	First visit	First GH stimulation test	Second GH stimulation test	Adult height
**Age (years)**	11.5	12.9	13.5	18.5
**Duration of treatment**				4.8 years
**IGF-1 (ng/mL)**	105	125	114	
**IGF-1 Z score (SD)**	−1.68	−2.06	−2.21	
**IGFBP-3 (mg/L)**		2.5	2.7	
**Standard IGFBP-3 values (mg/L)**		2.1–6.2	2.1–6.2	
**Testosterone total (ng/dL)**		12	79	
**Peak growth hormone (ng/mL)**		16.9	5.4	
**Height (cm)**	143.6	145	148.6	180.5
**Height Z score (SD)**	−0.67	−1.3	−1.65	0.57
**Change in height Z score during treatment (SD)**		−0.63	−0.98	+2.22
**Height velocity (cm/yr)**	3.8		2.8	
**Bone age (years)**	11.5	12	12.5	

Abbreviations: GH, growth hormone; IGF-1, insulin-like growth factor 1; IGFBP-3, insulin-like growth factor-binding protein 3.

He presented to us at age 13.5 years. His height had decreased to the 6th percentile (−1.52 SD).

### Case 2

**Figure 2.**
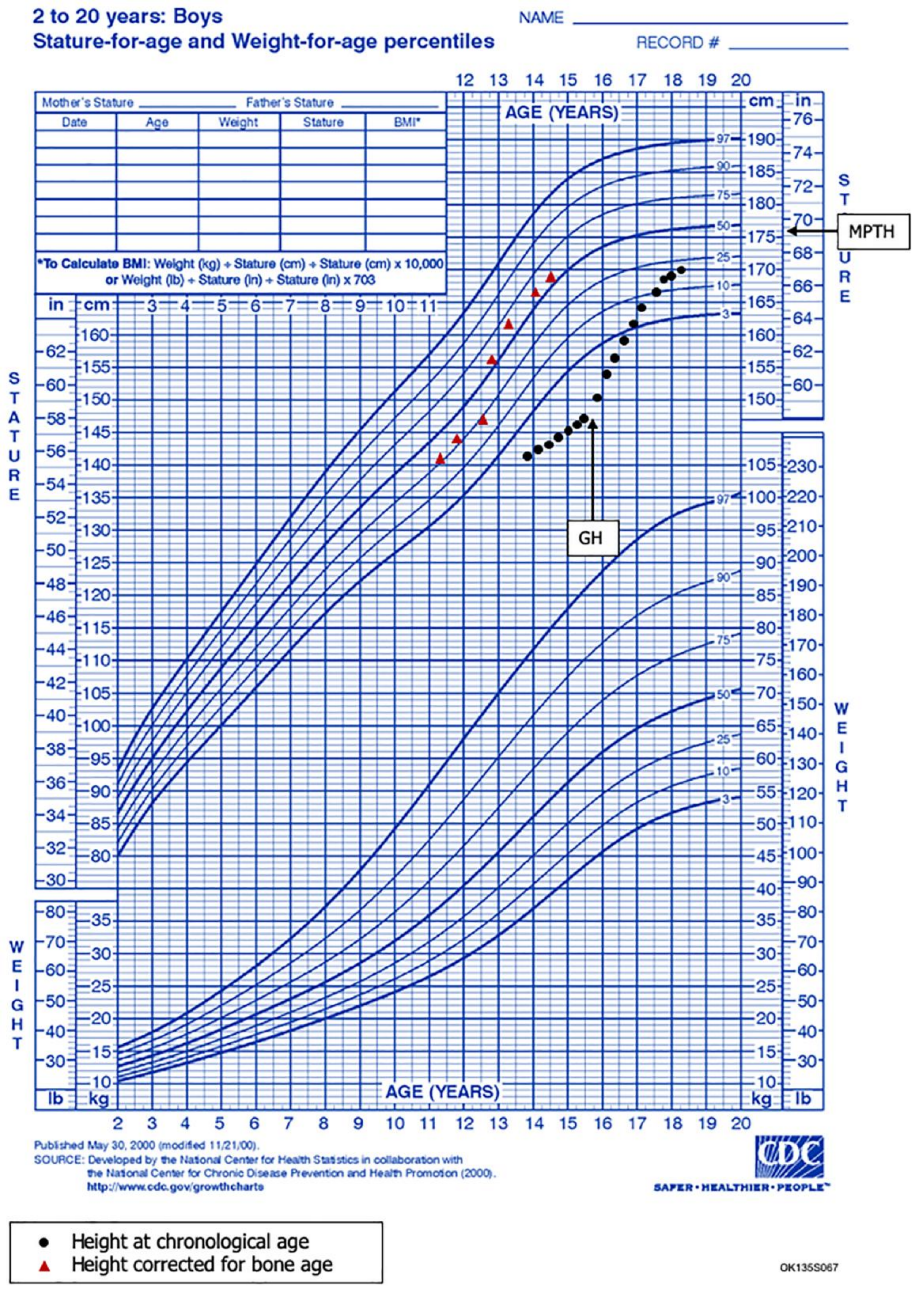
Patient 2 Growth Chart. Longitudinal growth chart in patient 2 before and after growth hormone treatment. GH, growth hormone; MPTH, Mid-Parental Target Height.

Patient 2 presented at 13.9 years for short stature with height of 141.2 cm at the 0.43 percentile (−2.63 SD; Fig. 2). His mid-parental target height was 175.7 cm (50th percentile).

The patient’s father started puberty at 12 to 13 years and his mother had menarche at 16.5 years of age. The family history includes an older brother diagnosed with GHD. The brother presented at 15.8 years with a height of 151.5 cm on the 0.43 percentile (−2.63 SD) and HV of 2.2 cm/year (−0.23 SD). At 16.3 years, he had a peak GH concentration 2.4 ng/mL (2.4 μg/L; arginine and L-dopa) with serum testosterone 37 ng/mL (1.28 nmol/L) and LH 0.6 mIU/mL (pubertal > 0.3 mIU/mL; 0.6 IU/L). He was treated with rhGH for 4 years and reached an adult of height of 176.8 cm, gaining 2.57 SD in height.

## Diagnostic Assessment

### Case 1

The patient’s BMI was at the 37th percentile, testicular volume 6 mL, and IGF-1 128 ng/mL (16.73 nmol/L; −2.02 SD for Tanner stage 2/3). His bone age was delayed by 1 year. When his height decreased further to the 5th percentile (−1.65 SD) and his HV was 2.84 cm/year in spite of advancing puberty, he underwent a repeat GHST with arginine and L-dopa. His peak GH concentration was 5.4 ng/mL (5.4 μg/L), consistent with GHD (Table 1). His serum testosterone of 79 ng/dL (2.74 nmol/L), luteinizing hormone (LH) of 3.2 mIU/mL (3.2 IU/L), and follicle-stimulating hormone (FSH) of 2.1 mIU/mL (2.1 IU/L) were consistent with puberty. His thyroid function tests were normal: TSH of 1.6 mIU/L (1.60μIU/mL; 0.8-3.9 uIU/mL); free T4 of 12.26 pmol/L (0.95 ng/dL; 0.60-1.10 ng/dL); total T3 of 2.53 nmol/L (164 ng/dL; 87-178 ng/dL); and total T4 of 100.6 nmol/L (7.8 ug/dL; 5.0-12.2 ug/dL). His 9:00 Am cortisol of 15.2 ug/dL (419.5 nmol/L) and prolactin of 10 ng/mL (10 μg/L) were normal. Pituitary magnetic resonance imaging was normal.

### Case 2

Patient 2 was prepubertal on initial exam. His BMI was at the 5th percentile. His IGF-1 was 163 ng/mL (21.3 nmol/L; −0.9 SD for Tanner stage I). His bone age was delayed 2.5 years. At 14.5 years, an unprimed GHST test with arginine and glucagon showed a peak GH of 19 ng/mL (19 μg/L; Table 2). The patient continued to be monitored without intervention. As puberty progressed during the next 9 months, testicular volume increased to 8 mL and testosterone to 83.9 ng/dL (2.91 nmol/L) while height decreased from −2.63 to −2.96 SD and HV was 3.8 cm/year (−0.12 SD). His BMI remained at the 5th percentile. His IGF-1 was 147 ng/mL (19.21 nmol/L; −2.4 SD for Tanner stage 2/3). He underwent a second GHST, also with arginine and glucagon, when his was LH 0.97 mIU/mL (0.97 IU/L) and DHEA-sulfate was 177 ug/dL (pubertal > 42 ug/dL; 4.6 nmol/L). His peak GH concentration was 9.2 ng/mL (9.2 μg/L), suggesting GHD (Table 2). His TSH was 2.58 mIU/L (2.58 uIU/mL; 0.45-4.5 uIU/mL); free T4 18.06 pmol/L (1.4 ng/dL; 0.93–1.60 ng/dL); total T4 108.36 nmol/L (8.4 ug/dL; 4.5–12 ug/dL); total T3 2.08 nmol/L (135 ng/dL; 75-180 ng/dL); cortisol 20 ug/dL (552 nmol/L); and prolactin 6.4 ng/mL (6.4 μg/L). The pituitary was small on magnetic resonance imaging, consistent with GHD.

**Table 2.** Patient 2

	First visit	First GH stimulation test	Second GH stimulation test	Most recent visit
**Age (years)**	13.9	14.5	15.3	17.1
**Duration of treatment**				2.2 years
**IGF-1 (ng/mL)**	163	113	130	275
**IGF-1 Z score (SD)**	−0.9	−2.1	−1.9	−0.2
**IGFBP-3 (mg/L)**	3.67	3.41	3.69	4.25
**Standard IGFBP-3 values (mg/L)**	2.53 - 6.20	2.58 - 6.27	2.61 - 6.31	2.66 - 6.32
**Testosterone total (ng/dL)**		16	83.9	
**Peak growth hormone (ng/mL)**		19	9.2	
**Height (cm)**	141.2	143.3	146.6	168.5
**Height Z score (SD)**	−2.63	−2.75	−2.96	−1.03
**Change in height Z score during treatment (SD)**		−0.12	−0.33	+1.93
**Height velocity (cm/yr)**		4.2	3.8	7.46
**Bone age (years)**	11.25	11.25	12.5	13.75

Abbreviations: GH, growth hormone; IGF-1, insulin-like growth factor 1; IGFBP-3, insulin-like growth factor-binding protein 3.

## Treatment

### Case 1

The patient was diagnosed with GHD after failing his second GHST. He was treated with recombinant human growth hormone (rhGH) of 0.14 mg/kg/week for 2 weeks before increasing to 0.28 mg/kg/week. He experienced no adverse events.

### Case 2

The patient was diagnosed with GHD after failing his second GHST. He was started on rhGH treatment of 0.12 mg/kg/week for 2 weeks then increased to 0.24 mg/kg/week. He experienced no adverse events.

## Outcome and Follow-up

### Case 1

The patient was treated uneventfully with daily rhGH for 4.8 years. At 18.5 years he reached an adult height of 180.5 cm (0.57 SD), 3.8 cm above his mid-parental target height and 2.7 cm above the father's adult height. He gained 2.22 SDs in height. His BMI was on the 36.8th percentile and he had no symptoms of adult GHD. His IGF-1 was 187 ng/mL (144-434 ng/mL; 24.44 nmol/L) and IGF-BP3 was 5.43 mg/L (2.68-6.33 mg/L) 3 to 4 months after the conclusion of GH treatment. Repeat GHST was not performed.

### Case 2

The patient showed significant improvements after starting rhGH treatment. His HV increased from 1.8 cm/year to 11.3 cm/year in 6 months. At 17.8 years, his height was 168.5 cm (−1.03 SD), HV was 7.5 cm/year, and bone age was delayed 3.5 years. His pubic hair was Tanner stage IV and testicular volume was 25 mL, with an afternoon testosterone level of 280 ng/dL (9.71 nmol/L). The patient has not yet reached adult height. He has gained 1.93 SD in height in 2.1 years of treatment (Table 2).

## Discussion

GHD can be defined as congenital or acquired, where congenital is due to genetic abnormalities and acquired is due to tumors, trauma, inflammation, brain infections or radiotherapy, but the majority of isolated GHD cases are idiopathic [7]. A recent systematic review by Hage et al described the potential mechanisms of GHD [7]. Zadik et al described late-onset GHD in 3 patients who initially tested GH sufficient, but after 3 to 4 years of longitudinal monitoring, upon retesting they were diagnosed with GHD [5]. Late loss of GH secretion has been described in patients with organic lesions and traumatic brain injuries [4]. The exact mechanism of idiopathic GHD remains unknown. We believe late-onset GHD is less likely to be due to anatomical changes and more likely due to functional transient decrease in somatomedin secretion.

Without monitoring the second patient's height longitudinally, the patients’ features were consistent with criteria for idiopathic short stature (ISS) with a height less than −2 SDS without evidence of pathology [8, 9]. The definition of ISS does not exclude constitutional delay of growth and puberty (CDGP) or familial short stature, considered by many as “normal variant short stature” [8, 9]. We recently recommended greater in-depth genetic evaluations along with longitudinal follow up to diminish the numbers of patients with growth failure of undetermined etiology, or ISS [9]. The second patient's initial height of −2.63 SD, lower than average IGF-1 levels, and delayed bone age led to a GHST test to which he showed a normal peak concentration, unprimed. However, because of longitudinal surveillance, we were able to diagnose GHD when his height and HV continued to decline despite pubertal progression. His prompt response to GH treatment further confirmed GHD.

Both patients had immediate accelerated growth after beginning rhGH treatment, which is the typical response for patients with GHD. On average, the HV of patients with GHD accelerates to 10 to 12 cm/year within the first year of treatment [1]. The HV of our patients improved from 2.8 cm/year and 1.8 cm/year to 10 cm/year and 11.3 cm/year, respectively, as expected for GH-treated patients with GHD. Furthermore, patients with CDGP can present with short stature but have normal HV for age and puberty, normal IGF-1 levels, and rarely reach expected adult heights [10]. Studies have shown that patients with IGF-1 SDS values of −2.0 or less are highly predictive of GHD [7]. Both patients described had low IGF-1 levels at 128 ng/mL (16.73 nmol/L; −2.02 SD for Tanner stage 2/3) and 147 ng/mL (19.21 nmol/L; −2.4 SD for Tanner stage 2/3) despite puberty. Based on the late-onset, evolving GHD in these patients, we recommend long-term follow-up in patients suspected of having ISS or CDGP. Similar to our patients, Zadik et al reported 3 patients with suboptimal growth rates and short stature with no organic pathology and normal initial GHST results, but repeat GHST 3 to 4 years later showed subnormal GH levels of 2.5 μg/L, 3.5 μg/L, and 1.9 μg/L, confirming the diagnosis of GHD [5].

GHSTs are known to have limitations, including poor reproducibility [7, 8]. Although we believe the features of the 2 cases are suggestive of GHD, it is possible that the results may be due to poor reproducibility. Furthermore, different agents were used for the initial and repeated GHST for the first patient; however, there are few published data on the impact of specific agents on GHST results. Current clinical recommendations require the use of 2 different agents but do not specify which should be utilized. Many clinicians classify GHD as a continuum from severe to normal, and with the development of more sensitive testing, some clinicians may use 5 or 7 μg/dL as the cutoff for GHD, although the currently accepted cutoff is still 10 μg/dL [2]. GHST still remains an essential part of GHD diagnosis in the context of the rest of the clinical picture [1, 3].

The Pediatric Endocrine Society recommends sex steroid priming in prepubertal boys 11 years and older before GHST to increase the specificity of testing and decrease the percentage of false-positive results [1]. However, sex steroid priming can also lead to false-negative results by increasing the amount of GH secreted [7]. The value of sex steroid–primed GHSTs to distinguish GHD from constitutional delay of growth and puberty is debated [8]. The patients we describe were moderately “self-primed” with increasing testosterone levels as puberty progressed, and on repeat GHST, they tested deficient. Retesting when testosterone levels were above 100 ng/dL would have strengthened the diagnosis of GHD and more clearly ruled out hypogonadism; however, we did not stop growth hormone treatment to retest.

Long-term follow-up and repeated testing was essential in diagnosing the evolving nature of these patients’ GHD. In both of these patients, the combination of careful clinical observation of decreasing height and height velocity as puberty progressed combined with selective screening tests such as IGF-1 prompted the performance of a repeat GHST that resulted in making the diagnosis of GHD. The prompt, excellent response to treatment along with long term monitoring during treatment substantiated the diagnosis in both. At 3 to 4 months after the conclusion of GH treatment, the first patient's IGF-1 was 187 ng/mL (144-434 ng/mL; 24.4 nmol/L). The second patient has not yet reached adult height, but puberty has progressed normally. At the most recent visit, his pubic hair was Tanner stage IV, testicular volume was 25 mL, and had an afternoon testosterone level of 280 ng/dL (9.7 nmol/L). Once reaching adult height, we will retest the second patient's IGF-1 levels, and if low, retest GH levels and consider treatment.

Without further evaluation and additional monitoring, these patients may have remained in the unexplained growth failure category, ultimately winding up as ISS. Late-onset GHD may be more common than is recognized in children with short stature prematurely diagnosed with ISS or CDGP. These patients highlight the importance of rigorous monitoring and repeat stimulation testing for patients with pubertal progression and persistent poor growth without a clear etiology.

## Learning Points

- Patients with short stature and “normal” growth hormone stimulation tests may be classified as idiopathic short stature without sufficient longitudinal follow-up.
- Isolated growth hormone deficiency can be diagnosed later in puberty via thorough evaluation using a combination of growth factors, bone age x-rays, and growth hormone stimulation tests.
- Longitudinal follow-up and evaluation of patients with short stature with repeat growth hormone stimulation test is essential to diagnose late-onset growth hormone deficiency, even if previous evaluations have been unyielding.

## Data Availability

Original data generated and analyzed during this study are included in this published article.

## Abbreviations

BMI: body mass index
CDGP: constitutional delay of growth and puberty
GH: growth hormone
GHD: growth hormone deficiency
GHST: growth hormone stimulation test
HV: height velocity
IGF-1: insulin-like growth factor 1
ISS: idiopathic short stature
LH: luteinizing hormone
rhGH: recombinant human growth hormone
TSH: thyrotropin (thyroid-stimulating hormone)
